# Supplementary material for: Predicting harmful alcohol use prevalence in Sub-Saharan Africa between 2015 and 2019: Evidence from population-based HIV impact assessment
Source: PLoS One. 2024 Oct 9;19(10):e0301735. doi: 10.1371/journal.pone.0301735 (PMC11463767; doi:10.1371/journal.pone.0301735)
Supplement: Attachment — Submitted filename: Response to Reviewers.docx [file pone.0301735.s001.docx]

Manuscript title: Predicting Harmful Alcohol Use Prevalence in Sub-Saharan Africa between 2015 and 2019: Evidence from Population-based HIV Impact Assessment

Dear Editor,

On behalf of all authors of “Predicting Harmful Alcohol Use Prevalence in Sub-Saharan Africa between 2015 and 2019: Evidence from Population-based HIV Impact Assessment” we sincerely thank you for the opportunity to resubmit our manuscript for consideration. We would also like to thank the reviewer whose thorough evaluation of our work has given us the opportunity to make substantive improvements.

Again, we are appreciative of this opportunity to resubmit our work to PLOS ONE. With the help of the academic editor and the reviewer, we believe we are resubmitting an improved text.

Sincerely,

Mtumbi on behalf of all authors

**Below is a point-by-point response to the concerns and comments of the Editor and Reviewer.**

**EDITOR’S COMMENTS TO THE AUTHOR**

**Comment [1]**

Background and Method Sections: Clearly define harmful alcohol use in both the background and method sections to ensure clarity for readers.

**Response**

*This has been addressed*

**Comment [2]**

Figure and Table Titles: Ensure that figure titles are placed below the figures, while table titles should be positioned above the tables for consistency and readability.

**Response**

*This has been addressed*

**Comment [3]**

Machine Learning Model Validation: Describe whether the machine learning model used in your analysis was validated. Additionally, indicate any limitations associated with the model used for this analysis.

**Response**

*This has been addressed by sentence 128 – 130*

*‘In this study, a confusion matrix and receiver operating characteristic (ROC) curve were used to assess algorithm performance using various metrics such as accuracy, sensitivity, specificity, F1 score, and area under the curve (AUC) (19) (20).’*

**Comment [4]**

Terminology: Ensure consistency in terminology usage. For instance, use "HIV" or “HIV/AIDS” instead of "HIV/ART".

**Response**

*The different terminologies were intentionally used to represent distinct stages and conditions related to HIV/AIDS. To ensure clarity and consistency, we define each term as follows:*

- *HIV: Refers to the actual infection*
- *HIV status: Describes the state of being either HIV-positive or HIV-negative.*
- *PLHIV refers to the state of contracting and having the HIV infection while remaining clinically stable.*
- *HIV/AIDS: Denotes the progression of HIV infection into the disease stage where symptoms and complications are present.*
- *HIV/ART: Indicates having an HIV infection and receiving antiretroviral therapy (ART).*
- *HIV/ART status: Refers to the state of being HIV-positive, with a distinction between those who are on enrolled ART and those who are not yet enrolled.*

**Comment [5]**

Definition of Excessive Alcohol Use: Clearly define what constitutes excessive alcohol use to provide readers with a clear understanding of this important terminology in your study.

**Response**

*We have edited to replace the term "excessive alcohol use" with "harmful alcohol use" for consistency.*

**Comment [6]**

Inclusion of Ethiopia in the Study: Acknowledge the absence of Ethiopia in the study population and discuss the implications of this omission. Additionally, if your study included findings on harmful alcohol use in Ethiopia, ensure that this is accurately reflected in the manuscript.

***Response***

*Ethiopia has been removed.*

**REVIEWER’S COMMENTS TO THE AUTHOR**

**Reviewer #1: General Comments**

1-The article would benefit from proofreading for grammatical errors, repetition, and clarity.

*This has been addressed*

2-Consideration should be given to the structure and organization of the article to improve flow and coherence.

*This has been addressed by inclusion of subheadings*

3- Citations should follow a consistent format throughout the article.

*This has been addressed*

**Major Comments**

**Comment [1]**

The abstract is repetitive, appearing three times with the same content. This should be corrected to provide a concise summary of the study objectives, methods, key findings, and implications.

**Response**

*This has been addressed*

**Comment [2]**

The introduction provides a good overview of the significance of harmful alcohol use and its association with HIV/AIDS globally and in SSA. However, it could be more concise and focused.

**Response**

*This has been addressed. The introduction and methods have been cut down and made to be more precise as before.*

**Comment [3]**

It would be helpful to clearly state the specific objectives of the study at the end of the introduction.

**Response**

*This has been addressed. Specific objectives have been included at the end of the introduction*

**Comment [4]**

The methods section lacks clarity in terms of the exact methodology employed for data analysis. It mentions the use of chi-square tests, logistic regression, and machine learning methods, but it doesn't provide details on how these were applied.

**Response**

*This been addressed.*

**Comment [5]**

Providing more information on the variables included in the analysis and justification for their selection would enhance the methodological clarity.

**Response**

*This has been addressed*

*Lines 105 – 109*

*The independent variables included age groups (in complete calendar years), sex (male/female), marital status (single, married, widowed and divorced), area of residence (rural/urban), highest education level (none, primary, secondary and tertiary), wealth index (poorest, poor, middle, rich and richest), country (stratified into Eswatini, Malawi, Namibia, Tanzania, Zambia and Zimbabwe), and HIV/ART status (negative, HIV+ not on ART and HIV- on ART).*

*Lines 96 – 104*

*Data management was done in R version 4.2. We merged data from six Sub-Saharan countries namely Eswatini, Malawi, Namibia, Tanzania, Zambia, and Zimbabwe. The primary outcome variable of interest was ‘harmful alcohol use’. ‘Harmful alcohol use’ in this context refers to patterns of drinking that result in adverse physical and psychological health outcomes (1). ‘Harmful alcohol use’ was assessed using the Alcohol Use Disorder Identification Test – C (AUDIT-C) based on the frequency of drinking, drinking category and quantity of drinking. The AUDIT-C used the three standard questions from module 10 of the PHIA questionnaire to generate the scores (11). The AUDIT-C is scored on a scale of 0-12 (scores of 0 reflect no alcohol use). Alcohol drinking is based on the AUDIT-C tool (AUDIT-C score ≤ 2 for women and ≤ 3 for men was classified as ‘harmful alcohol use’ (no); AUDIT-C score ≥ 3 for women and ≥4 for men was classified as ‘harmful alcohol use’ (yes).*

**Comment [6]**

The data management process is described briefly; providing more detail on how missing data were handled and how variables were recoded would improve transparency.

**Response**

*The model used in the analysis considered only complete observation and all observations with missing data were excluded.*

**Comment [7]**

It's important to specify the criteria used for harmful alcohol use classification based on AUDIT-C scores.

**Response**

*For classification purposes, AUDIT-C scores were interpreted as follows: a score of ≤ 2 for women and ≤ 3 for men was classified as 'harmful alcohol use: no'; a score of ≥ 3 for women and ≥ 4 for men was classified as 'harmful alcohol use: yes'. This scoring approach allowed us to differentiate between harmful and non-harmful alcohol use in the study population.*

*Clarification in Methods Section:*

*The primary outcome variable of interest was 'harmful alcohol use'. In this context, harmful alcohol use refers to patterns of drinking that result in adverse health outcomes, both physical and psychological. We assessed harmful alcohol use using the Alcohol Use Disorder Identification Test – C (AUDIT-C), which is based on the frequency of drinking, drinking category, and quantity of drinking. The AUDIT-C consists of three questions from module 10 of the PHIA questionnaire, generating a score on a scale of 0-12 (with scores of 0 indicating no alcohol use).*

**Comment [8]**

The section on ethical considerations should be moved to the beginning of the methods section.

**Response**

*Thank you, this has been addressed. Ethical consideration has been moved to the beginning of the methods section.*

**Comment [9]**

The results section is detailed and provides a comprehensive overview of participant characteristics and prevalence rates. However, it's quite lengthy and could be condensed

**Response**

*This has been addressed. Result section has been condensed*

**Comment [10]**

The presentation of results would benefit from clear subheadings and tables to improve readability.

**Response**

*Thank you, this has been addressed. Each set of tables and figures has a subheading under which they are falling*

**Comment [11]**

The discussion of factors associated with harmful alcohol use could be more structured, perhaps separated into subsections based on demographic and health-related variables

**Response**

*This has been addressed.*

**Comment [12]**

The discussion should focus on interpreting the findings in relation to existing literature, explaining the implications of the results, and discussing limitations and future research directions.

**Response**

*This has been addressed.*

**Comment [13]**

There's an opportunity to explore potential mechanisms underlying the observed associations and to discuss the implications for public health Interventions-It would be helpful to compare the findings of this study with previous research on harmful alcohol use and HIV/ART status in SSA.

**Response**

*We acknowledge the importance of exploring potential mechanisms underlying the observed associations and discussing the implications for public health interventions. However, it is important to note that, to the best of our knowledge, there have been no previous studies specifically focusing on the relationship between harmful alcohol use and HIV/ART status in Sub-Saharan Africa (SSA).*

**Comment [14]**

The Authors should summarize the key findings of the study and their implications for public health practice and policy and Suggestions for future research based on the study's limitations and unanswered questions should be provided.

**Response**

*This has been addressed.*
